# The Neuron Navigators: Structure, function, and evolutionary history

**DOI:** 10.3389/fnmol.2022.1099554

**Published:** 2023-01-12

**Authors:** Regina M. Powers, Robert F. Hevner, Shelley Halpain

**Affiliations:** ^1^Department of Neurobiology, School of Biological Sciences, University of California, San Diego, La Jolla, CA, United States; ^2^Sanford Consortium for Regenerative Medicine, La Jolla, CA, United States; ^3^Department of Pathology, UC San Diego School of Medicine, University of California, San Diego, La Jolla, CA, United States

**Keywords:** neuron navigator, neuritogenesis, macropinocytosis, actin, microtubules, growth cone, cell migration, axon guidance

## Abstract

Neuron navigators (Navigators) are cytoskeletal-associated proteins important for neuron migration, neurite growth, and axon guidance, but they also function more widely in other tissues. Recent studies have revealed novel cellular functions of Navigators such as macropinocytosis, and have implicated Navigators in human disorders of axon growth. Navigators are present in most or all bilaterian animals: vertebrates have three Navigators (NAV1-3), *Drosophila* has one (Sickie), and *Caenorhabditis elegans* has one (Unc-53). Structurally, Navigators have conserved N- and C-terminal regions each containing specific domains. The N-terminal region contains a calponin homology (CH) domain and one or more SxIP motifs, thought to interact with the actin cytoskeleton and mediate localization to microtubule plus-end binding proteins, respectively. The C-terminal region contains two coiled-coil domains, followed by a AAA+ family nucleoside triphosphatase domain of unknown activity. The Navigators appear to have evolved by fusion of N- and C-terminal region homologs present in simpler organisms. Overall, Navigators participate in the cytoskeletal response to extracellular cues *via* microtubules and actin filaments, in conjunction with membrane trafficking. We propose that uptake of fluid-phase cues and nutrients and/or downregulation of cell surface receptors could represent general mechanisms that explain Navigator functions. Future studies developing new models, such as conditional knockout mice or human cerebral organoids may reveal new insights into Navigator function. Importantly, further biochemical studies are needed to define the activities of the Navigator AAA+ domain, and to study potential interactions among different Navigators and their binding partners.

## Introduction

During organismal development, countless cell types integrate extra- and intracellular cues to form functional organs. These cues determine the destination of migrating cells, the morphology and genomic programming of differentiating cells, and the formation of networks, such as the synaptic network of the brain. While nearly all cell types undergo morphogenesis during development, the transformation of neural progenitor cells into polarized, functionally integrated neurons is especially complex, and critical to neural circuit function. This process includes cellular sphere symmetry breaking for neurite initiation, neurite elongation, axon/dendrite polarity specification, and the formation of the axonal growth cone—a cytoskeleton-rich structure that transduces guidance cues to direct the axon to its targets (McCormick and Gupton, 2020; Dorskind and Kolodkin, 2021; Zang et al., 2021). The cytoskeleton, which is comprised of microtubules, intermediate filaments, and actin filaments, undergoes major morphological rearrangements throughout these events, particularly at the cell periphery. Microtubules and actin filaments and their interactions have been a major focus of study in the context of early neuronal morphogenesis (Suter and Forscher, 2000; Dehmelt and Halpain, 2004; Dent et al., 2011; Bearce et al., 2015; Cammarata et al., 2016). During early development neurons respond to attractive and repulsive guidance cues to direct their migration and outgrowth, and cytoskeleton-associated proteins are important signal integrators and regulators of these processes.

### Cytoskeletal functions are critical in neural development

Dysregulation of the cytoskeleton underlies many disorders of human neurodevelopment (Pilz et al., 1998; des Portes et al., 1998a,b; Bellenchi et al., 2007). For example, mutations in several tubulin genes have been implicated in a group of neurodevelopmental disorders termed “tubulinopathies” (Gonçalves et al., 2018; Romaniello et al., 2018). Moreover, mutations in the microtubule associated proteins, Lis1 and DCX, cause cortical malformations resulting in lissencephaly and heterotopia, respectively, due to aberrant microtubule-dependent neuronal migration (Pilz et al., 1998; Jheng et al., 2018). Actin-related pathways are also commonly implicated in neurodevelopmental disorders. Mutations in SHANK3, a post-synaptic scaffolding protein that links glutamate receptors to the actin cytoskeleton, causes Phelan-McDermid Syndrome, a neurodevelopmental disorder associated with autism and intellectual disability (Wilson et al., 2003; Oberman et al., 2015). Another example is the Rho family guanine nucleotide exchange factor Trio, which controls the actin-regulation activities of Rac1 and Cdc42 (Estrach et al., 2002; Briançon-Marjollet et al., 2008) and is a risk gene for autism (Tian et al., 2021). Additionally, mutations in guidance cues can result in neurodevelopmental disorders *via* their impact on cytoskeleton-mediated events. For example, *reelin* mutations are associated with various neurodevelopmental conditions, including lissencephaly and autism (Hong et al., 2000; Fatemi, 2002; Chang et al., 2007). Reelin is a secreted glycoprotein that influences actin dynamics *via* the actin regulating protein cofilin (Chai et al., 2009), and interacts with the Lis1 signaling pathway *via* phosphorylation of Dab1, a Lis1 binding partner (Assadi et al., 2003). Loss of *reelin* signaling results in aberrant neuronal migration and neurite outgrowth (Chai et al., 2009; Frotscher et al., 2017). Other proteins that regulate cytoskeletal function have also been implicated in human neurodevelopmental disorders. For example, the regulatory/adaptor molecule 14–3-3ε, encoded by the YWHAE gene, and the protein Crk, encoded by the CRK gene, function downstream of reelin and regulate Lis1 function in neuronal migration (Assadi et al., 2003). These genes occur within the same 17p13.3 chromosomal region as the Lis1 gene (*PAFAH1B1*) that is frequently subject to microdeletion or microduplication events causative for the neurodevelopmental disorder Miller-Dieker Syndrome, in which patients exhibit severe lissencephaly (Cardoso et al., 2003; Toyo-oka et al., 2003; Yingling et al., 2003). Collectively, these studies stress the influence that signaling and cytoskeletal dynamics play in early brain development.

### Growth cones transduce external signals to establish neural circuitry

The growth cone is a specialized cytoskeleton-rich structure that responds to extracellular cues and directs neurite outgrowth. The growth cone has a stereotypical cytoskeletal arrangement that can be divided into three domains: the central domain is filled with microtubules, the peripheral domain is composed of F-actin that form filopodia and lamellipodia, and the transition zone is in between the central and peripheral domains where actin arcs and actin-enriched membrane ruffles form and where F-actin and microtubules can interact (Forscher and Smith, 1988; Forscher et al., 1992; Lin and Forscher, 1993; Pacheco and Gallo, 2016). The growth cone also contains many guidance cue receptors that signal to the cytoskeleton to direct neurite outgrowth toward the correct target *via* attractive guidance cues and away from incorrect targets *via* repellent guidance cues (Tessier-Lavigne and Goodman, 1996; Kalil and Dent, 2005; McCormick and Gupton, 2020; Dorskind and Kolodkin, 2021). While the growth cone has been extensively studied, numerous signaling pathways and mechanisms underlying growth cone morphology and behavior remain uncharacterized.

### Neuron Navigators

One understudied group of cytoskeletal proteins is the Neuron Navigator family. In vertebrates these are a family of three proteins (Neuron Navigator 1, 2, and 3) with potential cytoskeleton-interacting and other functional domains. Single gene homologs of the Navigators are also found in invertebrate species. For all organisms in which they have been investigated, Navigators are implicated in the development and morphogenesis of various cell types, and are especially important in neural development.

All known Navigator isoforms across species contain a AAA+ ATPase (AAA+) domain close to their C-terminus, and, although the function of this domain remains uncharacterized as of this writing, its evolutionary conservation suggests it is likely to represent the defining feature of the Navigator family. AAA + -domain containing proteins are, by definition, ATPases associated with diverse cellular activities, and it will be essential to characterize this property of the Navigators in order to fully understand their cellular functions.

Navigators 1 and 2 are important in neurite outgrowth, although the underlying mechanisms are still unclear. Despite the role that these proteins play in early development, there are relatively few studies addressing their function within the cell. This review will provide comprehensive information of what is currently known about the neuron Navigators and their homologs, and point out crucial gaps in our knowledge. Our group has recently provided evidence that Nav1 functions in macropinocytosis in the growth cone. Macropinocytosis is a form of fluid-phase endocytic uptake that occurs in many cells and regulates cell migration, neurite outgrowth, and synapse formation, among other activities. Here, we suggest that the regulation of macropinocytosis could account for most of the described morphogenic roles attributed to Navigator proteins.

## Initial discovery of the Navigators and their homologs

### Invertebrates

Invertebrate homologs of the neuron Navigators are *unc-53* in *Caenorhabditis elegans* and *sickie in Drosophila melanogaster*. By amino acid sequence, these invertebrate homologs most closely resemble Nav2. Navigator phenotypes associated with *unc-53* were first described after a screen for cell and axon migration mutants in *C. elegans* in 1987 (Hedgecock et al., 1987), and the function of the protein was described in more detail (Hekimi and Kershaw, 1993; Stringham et al., 2002). Later, *sickie* was identified by an RNAi screen in the fruit fly looking for genes involved in the immune system (Foley and O'farrell, 2004), and it subsequently was discovered to be involved in actin regulation during mushroom body development in the fly brain (Abe et al., 2014). More details regarding the function of these genes are provided below.

### Vertebrates

All three mammalian Navigators were identified and cloned in 2002 by various labs (Coy et al., 2002; Ishiguro et al., 2002; Maes et al., 2002; Merrill et al., 2002). Because multiple labs cloned the same genes, for a brief time the Navigators were referred to by multiple names, before “Neuron Navigators” became the consensus name for the protein family. It is important to emphasize, however, that these proteins are expressed in and function in multiple cell types, and here we will refer to them simply as the Navigators.

Nav3 was first called pore membrane and/or filament interacting like protein 1 (POMFIL1) by Coy *et al* (Coy et al., 2002) because after cloning and raising an antibody to the protein, they found it was present in nuclear pores in neurons *via* immunolabeling and electron microscopy. This group also cloned Nav1 and Nav2, which they called POMFIL3 and POMFIL2, respectively (Coy et al., 2002).

Nav2 was identified as an all-*trans* retinoic acid-responsive gene in the human neuroblastoma line SH-SY5Y, and named retinoic acid inducible in neuroblastoma cells (RAINB1; Merrill et al., 2002). That same year, Nav2 was identified in a search for genes that respond to adenomatous polyposis coli (APC), a transcription factor implicated in colorectal cancer^6^, and was called helicase APC down-regulated 1 (HELAD1; Ishiguro et al., 2002).

The most detailed and comprehensive previous study that cloned and characterized the mammalian Navigators was by Maes *et al* (Maes et al., 2002). This study cloned human Nav1 and described and compared amino acid sequences, alternative splicing, and tissue expression of all three Navigators (Maes et al., 2002), as well as compared the sequences to mouse Nav1, and Sickie and unc-53 proteins in *D. melanogaster* and *C. elegans*, respectively. These authors also investigated the phylogenetic relationships and evolution of the Navigator genes, and concluded that Navigators are conserved from nematodes through mammals. *Drosophila* melanogaster (Maes et al., 2002) and multiple fish species including zebrafish (as identified through NCBI BLAST search), all express related genes having a homologous AAA+-domain containing C-terminus. The authors demonstrated that Nav2 and Nav3 are more closely related to each other than to Nav1 (Maes et al., 2002). Human Nav1, Nav2, and Nav3 are found at chromosomal locations 1q32.1, 11p15.1, and 12q21.1, respectively, and genomic analysis suggested that a duplication event from the ancestor yielded two branches, both of which underwent a second duplication, which yielded Nav2 and Nav3 from one branch and Nav1 from the other. Maes et al. also pointed out that it is likely that Nav1 lost its 5′ end (encoding the CH domain) during one of these genomic rearrangements (Maes et al., 2002).

## Navigators have conserved domains and extensive disordered regions

**Figure 1:**
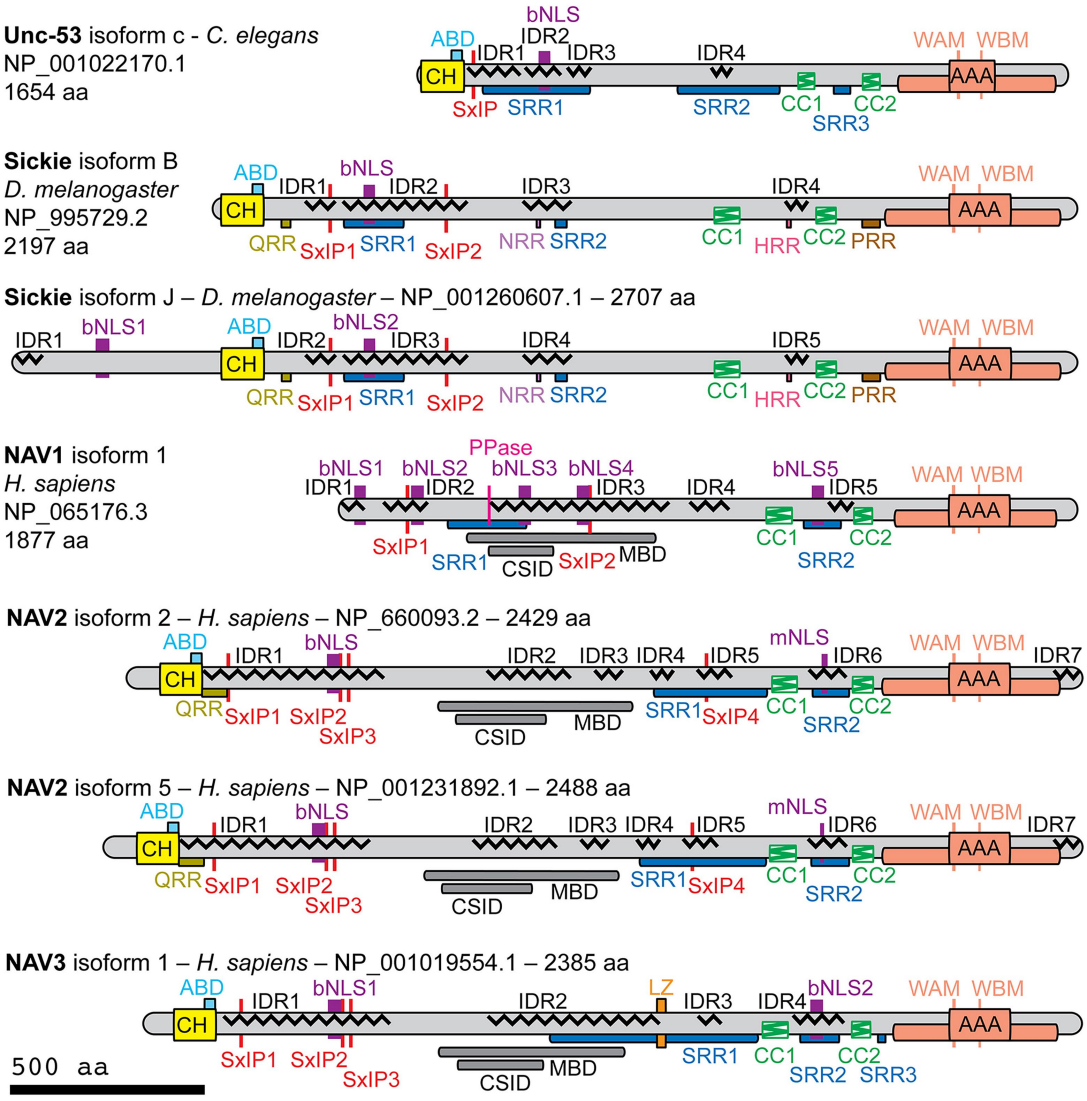
Structural features of NAV family proteins as determined by protein sequence analysis. Indicated isoforms of Unc-53 (*Caenorhabditis elegans*), Sickie (*Drosophila melanogaster*), and human NAV1, NAV2, and NAV3 were analyzed using protein sequence algorithms. The catalytic core of each AAA+ domain is indicated by the central box, with additional conserved domain components indicated by the surrounding box. Abbreviations: ABD, actin-binding domain (putative); bNLS, bipartite nuclear localization sequence; CC, coiled-coil; CH, calponin homology; CSID, cytoskeletal-interacting domain (putative); HRR, histidine-rich region; IDR, intrinsically disordered region; LZ, leucine zipper (putative); MBD, microtubule-binding domain (putative); mNLS, monopartite nuclear localization sequence; PRR, proline-rich region; PPase, inorganic pyrophosphatase (putative); QRR, glutamine-rich region; NRR, asparagine-rich region; SRR, serine-rich region; WAM, Walker A motif; WBM, Walker B motif.

Previous studies found that Navigators and their invertebrate homologs have AAA+ nucleotide triphosphatase (NTPase), calponin homology (CH), and coiled-coil (CC) domains, with the exception that NAV1 lacks the CH domain, likely due to secondary loss (Maes et al., 2002; Peeters et al., 2004; Stringham and Schmidt, 2009; Abe et al., 2014; Accogli et al., 2022). However, those differ in the number of reported CC domains (2, 3, or 4), and in the reported presence or absence of conserved microtubule- or cytoskeletal-binding domains. To resolve these discrepancies and further investigate the structure of Navigator family proteins, we here used current protein sequence analysis tools to study human (h) NAV1, hNAV2, and hNAV3; as well as NAV homologs Unc-53 (*C. elegans*) and Sickie (*D. melanogaster*). Since each protein has multiple potential isoforms generated from different reported transcript variants, we focused our analysis on well-documented isoforms with NCBI CCDS (consensus cDNA sequence) or Ensembl Canonical designations. The isoforms and details of sequence analysis are indicated in Figure 1, and in Supplementary Excel Workbooks 1–6.

Navigator family proteins are relatively large (>200 kD) and overall basic proteins (pI 8.2–9.5). With the exception of NAV1 (which lacks the CH domain), Navigators contain an N-terminal CH domain (~110 aa), and a C-terminal AAA+ domain (Figure 1; Supplementary Excel Worksheets S1–S5). Navigator CH domains were previously proposed to contain a single actin binding domain (Coy et al., 2002), but more recent studies suggest that CH domains typically contain multiple actin-binding sequences (Yin et al., 2020). The AAA+ domains have a core ATPase region (116–155 aa) identified by sequence analysis, flanked by extended AAA+ domain structures (401–452 total aa) inferred by structure prediction with AlphaFold (Jumper et al., 2021) and conservation analysis with COBALT (NCBI; see below). As determined by DeepCoil2 (Zimmermann et al., 2018), each protein also contains two CC domains (each between 40 and 70 aa), in conserved locations proximal to the AAA+ domain (Figure 1). We also found that Navigator family proteins contain extensive low complexity sequences (LCSs), such as serine-rich regions (SRRs), and numerous predicted intrinsically disordered regions (IDRs), with LCSs often overlapping IDRs (Figure 1).

Additional motifs predicted in Navigator family proteins included monopartite (mNLS) and bipartite (bNLS) nuclear localization sequences (NLSs; Figure 1; Lu et al., 2021), which target proteins for nuclear import (Figure 1). However, only NAV1 contains an NLS in the first 60 amino acids, where NLSs are most effective. NLS predictions do not necessarily indicate functional efficacy, but typically reflect highly basic sequences (Pemberton and Paschal, 2005). Since Navigators (including NAV1) are predominantly associated with cytoskeletal structures outside the nucleus, such as axons and growth cones, the significance of NLS motifs in Navigator family proteins remains uncertain.

Contrary to previous research reporting a “highly conserved” microtubule-binding domain (MBD) between the CH and CC domains (Peeters et al., 2004), the present analysis found no known consensus MBDs in Navigator family proteins (Figure 1). The putative MBD identified in NAV1 by deletion analysis (Martinez-Lopez et al., 2005) showed only low homology to the corresponding regions of NAV2 (36% identity) and NAV3 (38% identity), and no significant homology to Sickie or Unc-53. Likewise, the putative MBD (also termed a cytoskeleton-interacting domain) of NAV2 (Muley et al., 2008) showed only low homology to the corresponding regions of NAV1 (35% identity) and NAV3 (50% identity), and no significant homology to Sickie or Unc-53. Notably, the putative MBD regions contained extensive IDRs. Since IDRs may play a key role in the assembly and disassembly of microtubules and actin filaments (Wright and Dyson, 2015), it seems plausible that the poorly conserved IDRs in Navigator family proteins may mediate interactions with cytoskeletal proteins. Our analysis found that other proposed interaction sequences, including putative actin-binding (LKK, polyproline) and SH3-binding (PXXP) motifs (Coy et al., 2002; Stringham et al., 2002; Abe et al., 2014), were not conserved among Navigator family proteins.

Another mode of interaction with microtubules is suggested by previous studies that identified Navigators as microtubule plus-end tracking proteins (+TIPs) that reorganize the cytoskeleton (Martinez-Lopez et al., 2005; van Haren et al., 2009; Sanchez-Huertas et al., 2020). Many +TIPs bind microtubule end-binding (EB) family proteins through hydrophobic SxIP motifs (Honnappa et al., 2009), which are present in Unc-53 (Figure 1, this paper), Sickie (Abe et al., 2014), NAV1 (Sanchez-Huertas et al., 2020), and NAV2 (Abe et al., 2014). To assess SxIP motifs in Navigators, we used criteria established by proteomic analyses (Jiang et al., 2012). Specifically, SxIP motifs are considered functional if they meet three criteria: (a) they have the canonical sequence [ST]-X-[IL]-P (Ser or Thr may be the first amino acid, and Ile or Leu may be the third amino acid); (b) they meet “SxIP-9AA” contextual criteria requiring the presence of at least one basic amino acid but no acidic amino acids; and (c) are found in an IDR (Jiang et al., 2012). These criteria identified one SxIP motif in Unc-53, two in Sickie, two in hNAV1, four in hNAV2, and three in hNAV3 (Figure 1). The SxIP motifs were invariably found in the N-terminal half of Navigators, although hNAV2 had an additional SxIP motif in the C-terminal half of the protein. A previously reported SxIP motif adjacent to the AAA+ domain (Abe et al., 2014) was not confirmed, because it contained an acidic amino acid and was not in an IDR.

**Figure 2:**
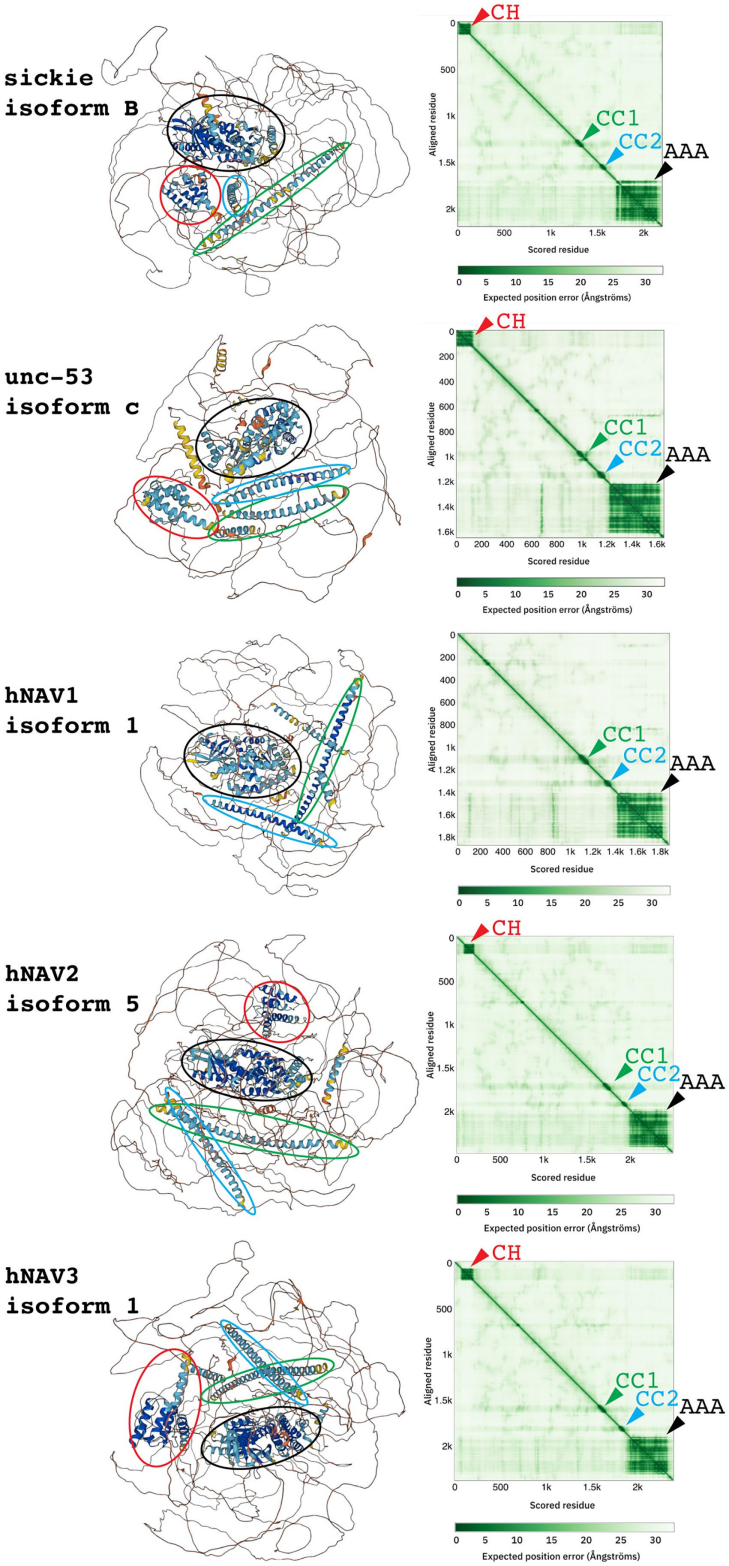
Three-dimensional structures (left) and predicted aligned error graphs (right) for indicated NAV family proteins. In each protein, four domains (CH, CC1, CC2, and AAA+) were identified, as indicated by colored ovals encompassing predicted structures, and matching color arrowheads on graphs. Between the four domains, structure predictions were low confidence.

Prediction of three-dimensional Navigator protein structures using AlphaFold (Jumper et al., 2021) confirmed and extended results from sequence analysis. Indeed, AlphaFold identified the CH domain (absent in NAV1), two CC domains, and the AAA+ domain in each human Navigator family protein (Figure 2). Interestingly, the AAA+ domains predicted by AlphaFold were substantially larger (~400–450 aa) than the core AAA+ NTPase domains predicted from sequence analysis (~110–160 aa), consistent with typical properties of AAA+ family proteins (Hanson and Whiteheart, 2005; Seraphim and Houry, 2020). Outside the major domains of Navigators, the predicted positions of amino acids were generally low confidence, consistent with the predominance of LCSs and IDRs. The low confidence of predicted inter-domain sequence positions could result from a lack of experimentally determined structures for similar sequences, although AlphaFold can accurately predict protein structures even when similar structures are unknown (Jumper et al., 2021). Thus, it is likely that putative IDRs in Navigator family proteins do not form stable 3-dimensional structures, but are instead flexible and dynamic.

**Figure 3:**
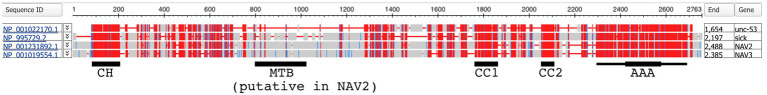
Alignment and conservation of protein sequences for Unc-53 isoform c, Sickie isoform B, hNAV2 isoform 5, and hNAV3 isoform 1. Highly conserved sequences are indicated in red, less conserved sequences in blue, and non-conserved sequences in gray. Conservation is based on the relative entropy threshold of residues, and does not indicate amino acid identity. Highly conserved sequences were observed for CH, CC1, CC2, and AAA+ domains. For AAA+ domains, the thick line indicates the NTPase core, and the thin line indicates the presumed entire domain. The putative microtubule-binding domain (MBD); also called the cytoskeletal-interacting domain) defined by deletion analysis of hNAV2 (Muley et al., 2008) was not conserved across NAV family proteins.

To examine the conservation of sequences and domains across Navigator family proteins, NCBI COBALT was used to align Unc-53, Sickie, hNAV2, and hNAV3 (Figure 3; hNAV1 was excluded because it lacks the CH domain.). This analysis showed that the CH, CC1, CC2, and AAA+ domains were highly conserved among these proteins, while intervening sequences were less conserved and had frequent insertions or deletions (Figure 3). The cytoskeletal-interacting domain of hNAV2 that was defined in deletion experiments (Muley et al., 2008) was not well conserved in other Navigators. Interestingly, the core AAA+ domain was flanked by highly conserved sequences spanning ~400-450aa, confirming that the extended AAA+ structural domain predicted by AlphaFold is indeed highly conserved across the Navigators.

**Figure 4:**
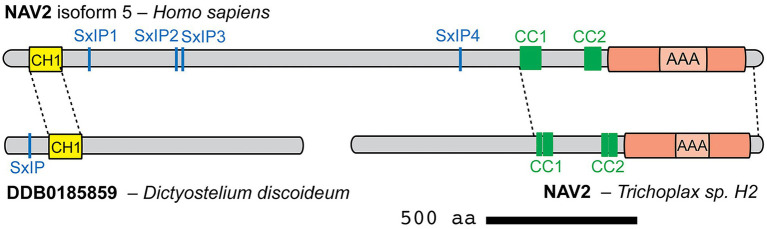
Comparison of human NAV2 (isoform 5) with NAV-related proteins from *Dictyostelium discoideum* and *Trichoplax* sp. *H2*. Homologous portions of each protein are indicated by dashed lines (identified by sequence aligment using BLAST). Canonical NAVs may have arisen by fusion of a N-terminal singlet CH1 protein with a C-terminal protein containing two CC and one AAA+ domains.

To determine how different transcript variants and isoforms might affect protein structure and potential interactions, we compared well-documented isoforms for several Navigator family proteins. For hNAV1, we compared the canonical isoform 1 (1877 aa) and the shorter isoform 2 (1,483 aa): alternative exon usage affected IDR and LCS sequences but did not alter CC or AAA+ domains. For hNAV2, we compared isoforms 2 (2,429 aa) and 5 (2,488 aa): the additional sequences in isoform 5 likewise affected LCS and IDR regions, but not CH, CC, or AAA+ domains (Figure 4). For Sickie, we compared isoforms B (2,197 aa) and J (2,707 aa): in this case, isoform J contained additional N-terminal protein sequences (including an IDR), but major domains (CH, CC, AAA+) were again not altered (Figure 4). From these examples, we conclude that different NAV isoforms mainly alter regions outside the major conserved domains, possibly to modulate cytoskeletal binding or other targeting interactions.

Together, the data indicate that Navigator family proteins, including their invertebrate homologs, have conserved AAA+, CC1, and CC2 domains, usually accompanied by an N-terminal region with multiple SxIPs and one CH domain (except in NAV1). These major domains are connected by extensive LCS and IDR sequences, which may serve to diversify interactions with the cytoskeleton or other targets. The AAA+ domain of Navigator family proteins belongs to the McrB family in the helix-2 insert clade of AAA+ proteins (Iyer et al., 2004). Interestingly, the McrB family AAA+ proteins use GTP rather than ATP as cellular energy source (Nirwan et al., 2019), suggesting that Navigator family proteins may be GTPases that function in cellular morphogenesis.

## Evolution of the Navigator family

So far, Navigators have been characterized in diverse species of protostome and deuterostome animals such as roundworm, insect, and human. Thus, Navigators were likely present already in the earliest bilaterian animals (~570 million years ago), and possibly earlier. However, it is unknown if any non-bilaterian species produce Navigators.

One putative Navigator-related protein, named DDB0185859 (protein sequence Q54Q34), has been described in a Protist, *Dictyostelium discoideum*, a species of slime mold (Friedberg and Rivero, 2010). DDB0185859 was characterized as having a single CH domain (CH1-type) related to that in Navigators. Sequence analysis using current algorithms confirmed that DDB0185859 has an N-terminal CH domain with homology to Navigators, but no coiled-coil or AAA+ domains were identified (Supplementary Figure S1). Thus, while the CH domain of DDB0185859 is related to the CH domain in Navigators, DDB0185859 does not itself qualify as a Navigator. Interestingly, an SxIP motif was also identified in DDB0185859, although it is unknown whether Protists utilize such motifs to interact with microtubules. Since *Dictyostelium discoideum* belongs to the kingdom Protista, it is likely that singlet CH1-type proteins evolved in the earliest eukaryotes (Friedberg and Rivero, 2010), and may have used SxIP motifs to interact with microtubules.

**Figure 5:**
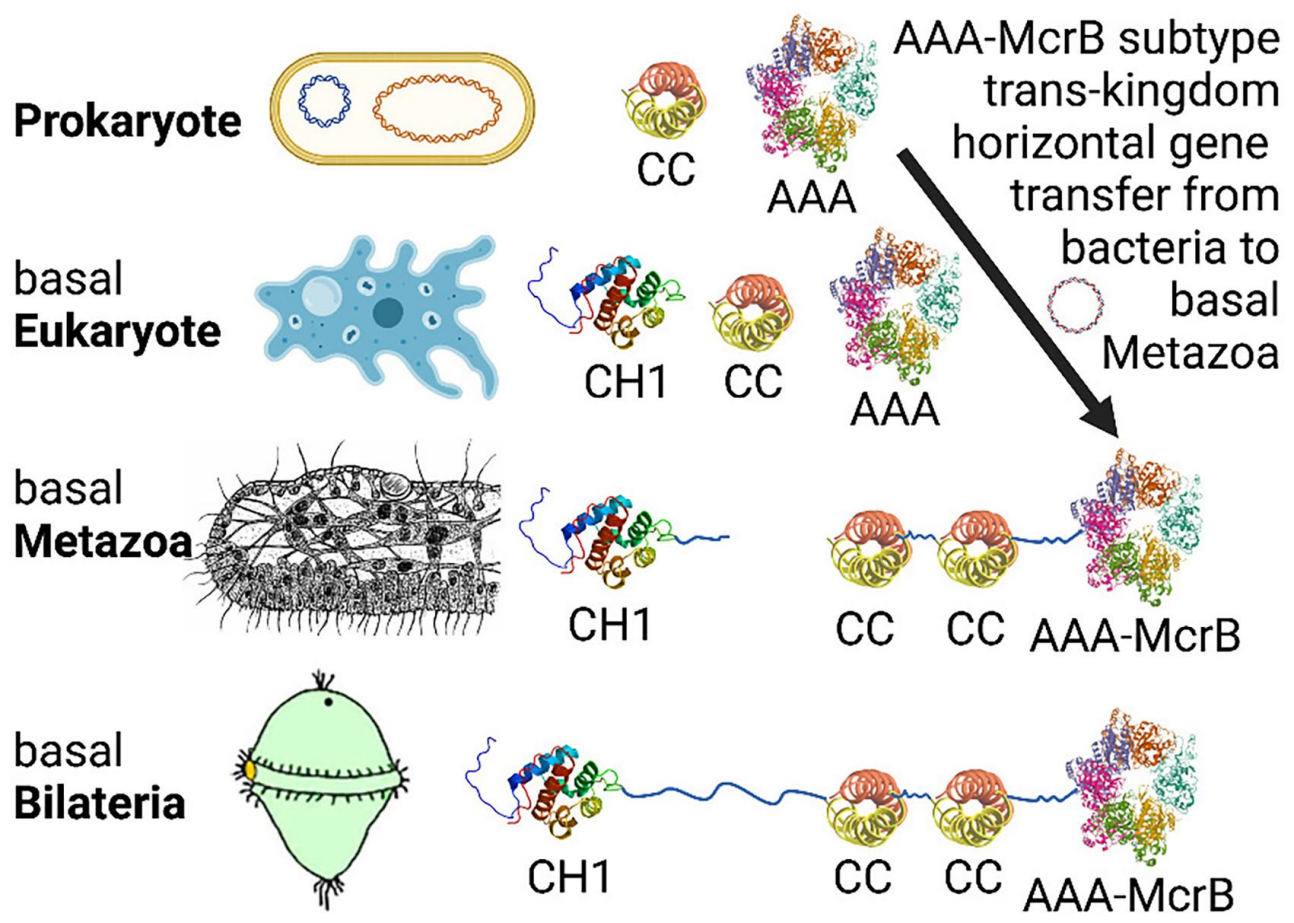
Proposed evolutionary history of bilaterian NAVs by domain recombination. The CC and AAA+ domains appear already in prokaryotes, while CH domains first appear and are ubiquitous among eukaryotes. The AAA + -McrB subtype was either lost from plants, or moved from bacteria to basal Metazoa by “trans-kingdom horizontal gene transfer” (Iyer et al., 2004), perhaps after feeding-related consumption. The C-terminal precursor region of NAVs (consisting of linked CC1, CC2, and AAA+ domains) was assembled in basal Metazoa, as indicated by the structure of the *Trichoplax* sp. *H2* NAV2-like protein. Finally, the N-terminal precursor region containing the CH1 domain (and SxIP motifs, not shown) was fused with the C-terminal precursor to form canonical NAVs in basal Bilateria. Interestingly, loss of the CH domain from NAV1, thought to have occurred after gene duplication in the lineage to vertebrates (Maes et al., 2002), makes NAV1 resemble the NAV-like C-terminal precursor as observed in *Trichoplax*. Figure produced using Biorender.

Searches in OrthoDB identified additional putative Navigator-related proteins in two groups of non-bilaterian animals: *Hydra vulgaris* (freshwater polyp) in the group Cnidaria, and *Trichoplax adhaerens* and *Trichoplax* sp. *H2* in the group Placozoa. The homologies and gene names were assigned by automated systems and are considered preliminary. In *Hydra*, the *LOC100207311 Neuron Navigator 1* gene encodes multiple isoforms, the longest of which is isoform X1 with 1,514 aa. Sequence analysis of isoforms X1 (XP_047126242.1) and X4 (XP_047126245.1) revealed a C-terminal AAA+ (P-loop NTPase) domain, but no CH or coiled-coil domains. In *Trichoplax adhaerens*, hypothetical protein TRIADDRAFT_54591 (785 aa) was tagged as “similar to neuron navigator 1.” Sequence analysis of TRIADDRAFT_54591 (XP_002111049.1) identified two coiled-coil domains, but no CH or AAA+ domains. In *Trichoplax* sp. *H2*, the *Neuron Navigator 2* (*NAV2*) gene encodes a 1,349 aa protein (RDD46365.1), which on sequence analysis was found to contain two CC domains and a C-terminal AAA+ domain (Supplementary Figure S1). The CC domains each consisted of two subdomains separated by only 4–6 aa (Supplementary Figure S1; Supplementary Excel Worksheet S6). By FASTA Protein Similarity Search, NAV2 of *Trichoplax* sp. *H2* was most closely related to the Navigator family based on sequences from vertebrate and invertebrate Navigators. Interestingly, the relative positions of the coiled-coil and AAA+ domains in NAV2 of *Trichoplax* sp. *H2* are similar as in NAVs of humans and other Bilateria (Figures 4, 5). Thus, *Trichoplax* sp. *H2* NAV2 resembles the C-terminal half of Navigator proteins in bilaterians, but lacks the CH domain present in most other Navigators. Indeed, *Trichoplax* sp. *H2* NAV2 resembles vertebrate NAV1 in lacking the CH domain, although NAV1 is thought to have lost the CH domain secondarily (Maes et al., 2002; Figure 5). Since *Trichoplax* belong to Placozoa, a basal eumetazoan clade that lacks neurons (Srivastava et al., 2008; Kamm et al., 2018), it appears that the C-terminal region of Navigators (containing two CC and one AAA+ domain) was present in early Metazoa (Figure 5).

In summary, proteins similar to Navigator N-terminal (containing the CH domain) and C-terminal (containing two coiled-coil and one AAA+ domains) regions have been identified in non-bilaterian species. However, no complete Navigator proteins have been recorded outside Bilateria. Interestingly, the McrB clade of AAA+ domains are posited to have undergone trans-kingdom horizontal gene transfer to eukaryotes from the ancestral prokaryotes from which they probably originated (Iyer et al., 2004). We speculate that complete Navigators formed by fusion of N- and C-terminal precursors, as a consequence of genome rearrangement around the time when Bilateria arose (Figures 4, 5).

### Transcriptional regulation of navigator genes during cerebral cortex development

The Navigators are expressed in many developing and adult mammalian tissues, including the central nervous system (Maes et al., 2002). However, little information is available regarding the transcriptional regulation of the Navigator genes. Interestingly, one region of the developing brain where specific transcription factors have been found to regulate Navigator gene expression is the embryonic mouse cerebral neocortex. Within this region, all three Navigators are expressed in the intermediate zone and cortical plate (Coy et al., 2002; Peeters et al., 2004; Martinez-Lopez et al., 2005; Pook et al., 2020; Powers et al., 2022), where newly generated cortical neurons migrate, differentiate, and grow axons and dendrites. Transcription factors Pax6, Tbr1, and Tbr2 (also known as Eomes) are important regulators of cortical neuron differentiation that also directly regulate Navigator gene expression (Englund et al., 2005; Mihalas and Hevner, 2017; Elsen et al., 2018). Pax6 binds the *Nav2* gene in embryonic neocortex *in vivo* (Pattabiraman et al., 2014; Ypsilanti et al., 2021), and expression of *Nav2* is profoundly reduced in *Pax6* null embryonic neocortex (Holm et al., 2007), suggesting that Pax6 directly activates *Nav2* gene expression, possibly by recruiting epigenetic factors that “unlock” *Nav2* (Elsen et al., 2018). Similar evidence indicates that Tbr2 and Tbr1 likewise strongly activate *Nav2* gene expression by direct binding (Bedogni et al., 2010; Elsen et al., 2013; Notwell et al., 2016; Sessa et al., 2017; Elsen et al., 2018).

The potent activation of *Nav2* by Pax6, Tbr2, and Tbr1 implicates *Nav2* as particularly important for cortical neuron development. Indeed, *Nav2* hypomorphic mutant mice have a small corpus callosum (a major cortical axon tract), and a human patient with *Nav2* mutations displayed cortical dysgyria (a neuronal migration disorder), as well as hypoplasia of the corpus callosum and anterior commissure, among other abnormalities (Accogli et al., 2022).

Tbr2 and Tbr1 also appear to directly regulate the *Nav1* gene, although the effects on *Nav1* expression are not as strong as for *Nav2*. Both Tbr1 and Tbr2 bind the *Nav1* gene (Notwell et al., 2016; Sessa et al., 2017). In *Tbr2* null neocortex, *Nav1* mRNA levels were slightly but significantly increased, suggesting that Tbr2 may directly repress *Nav1* expression (Elsen et al., 2013, 2018). In contrast, *Nav1* mRNA levels were moderately reduced in *Tbr1* null neocortex, suggesting that Tbr1 activates *Nav1* expression for cortical neuron development (Bedogni et al., 2010). Consistent with this interpretation, Nav1 protein is highly expressed in the axons of cortical neurons *in vivo* (Powers et al., 2022). Although *Nav3* is also expressed in developing cerebral cortex (Coy et al., 2002), this gene does not appear to be significantly regulated by Pax6, Tbr2, or Tbr1.

Together, the previous studies suggest that *Nav* genes are important for cerebrocortical neuron migration and axon growth. Moreover, *Nav1* and *Nav2* are regulated by key transcription factors in cortical development.

## Tissue expression and intracellular localization of Navigators

### Tissue expression

In invertebrates and vertebrates, the Navigators are expressed in multiple tissues, and are most highly expressed during development. In *C. elegans*, *unc-53* is expressed in sex myoblasts, excretory canal cells, and certain neurons (Stringham et al., 2002). Multiple studies used Northern blot analysis to identify tissue-level expression of the Navigators in vertebrates. Consistently, all three Navigators are expressed in the developing brain, but the mRNA for the three Navigators are expressed at different levels in different tissue such as the heart, lungs, liver, and skeletal muscle, which may indicate distinct functions for each Navigator isoform (Coy et al., 2002; Maes et al., 2002). Klein *et al* characterized *nav3* in zebrafish, finding that it is expressed in the endoderm and regulates the developing liver (Klein et al., 2011). Notably, the roles of the Navigators in non-brain tissues remain largely unexplored. Furthermore, splice variants for each Navigator may be differentially expressed compared to the full length transcript.

**Figure 6:**
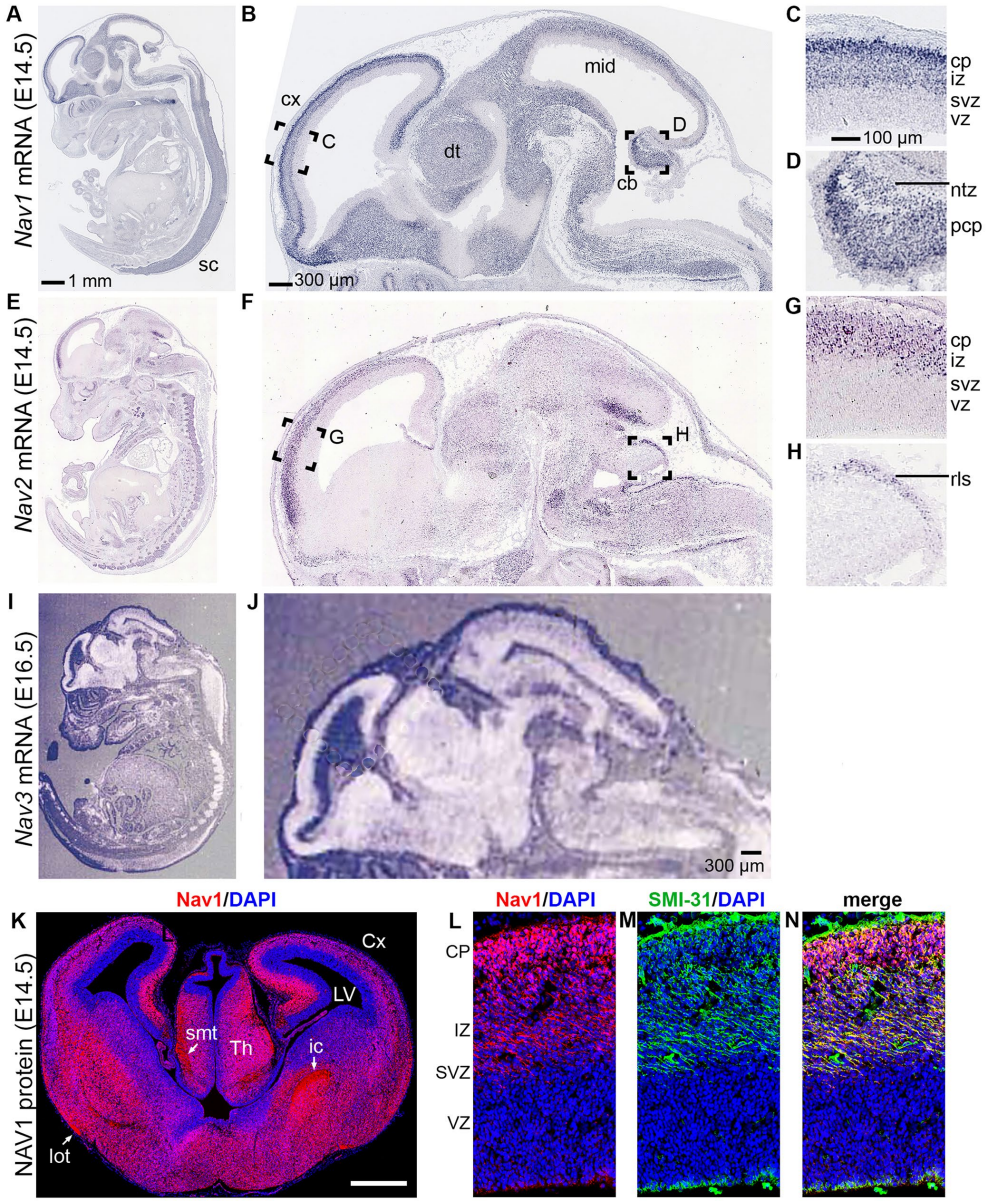
Navigator expression in the CNS. **(A–D)** Nav1 mRNA expression from GenePaint. **(E–H**) Nav2 mRNA expression from GenePaint. **(I–J)** Nav3 mRNA expression (Reproduced with permission from Coy et al., 2002; Note that the lower resolution of the original Nav3 image precludes detailed examination of cell layers). **(K–N**) Nav1 protein expression (red) with DAPI (blue) to label nuclei and SMI-131 (green) to label axons. Labeling demonstrates Nav1 expression in neurons and axons in the upper layers of developing cortex (Reproduced with permission from Powers et al., 2022). sc, spinal cord; cx, cortex; dt, dorsal thalamus; mid, midbrain; cb, cerebellum; cp, cortical plate; iz, intermediate zone; svz, subventricular zone; vz, ventricular zone; Cx, cortex; LV, lateral ventricle; rls, rostral rhombic lip migration stream; Th, thalamus.

Previous studies using *in situ* hybridization, as well as databases such as GenePaint (Visel et al., 2004) and the Allen Brain Atlas (Thompson et al., 2014), have revealed different patterns of gene expression for the different *Nav* family members in the developing mouse CNS (Figure 6). In mouse embryos, *Nav1* is widely expressed in neuronal differentiation zones of the brain and spinal cord (Martinez-Lopez et al., 2005). *Nav2* shows more restricted expression in regions such as the cerebral cortex, lateral geniculate nucleus of thalamus, and cerebellar rhombic lip migration stream (Peeters et al., 2004; Pook et al., 2020). Moreover, the full-length *Nav2* transcript is the predominant splice isoform during late embryonic and early-postnatal brain development. *Nav3*, like *Nav1*, also appears to show widespread expression in neuronal differentiation zones of the embryonic mouse brain (Coy et al., 2002).

The only Navigator protein studied by immunohistochemistry *in vivo* so far is NAV1, which is expressed in growing axon tracts, soma, and dendrites within developing neocortex (Powers et al., 2022; Figure 6). These observations are consistent with the conclusion that Navigators are important for axon guidance and neurite development, as indicated by studies of mice and humans with Navigator mutations.

Overall, Navigators appear to function during periods of extensive tissue morphogenesis, suggesting this may be one of their most essential roles. However, this information comes from a small number of studies, and more research is required to further characterize the expression and function of Navigators in different tissues.

### Subcellular localization

The subcellular localization of Navigators has been best characterized using vertebrate neurons in primary cultures and in neuroblastoma and other cancer-derived cell lines. All three mammalian Navigators partially localize to microtubule plus ends (van Haren et al., 2009, 2014; Sanchez-Huertas et al., 2020; Powers et al., 2022), and Nav1 and Nav2 were shown to use SxIP motifs to mediate this localization. Because Nav3 also contains SxIP motifs (Figure 1), it is assumed Nav3 plus tip localization is also mediated *via* SxIP domains. However, the Navigators are not exclusively located at plus ends; they are also found elsewhere in the cytoplasm, sometimes, but not always, associated with particular cellular structures (van Haren et al., 2009; Powers et al., 2022).

The intriguing suggestion that Nav3/POMFIL is detected at nuclear pores has not so far received further experimental attention, but would perhaps align with the observed NLS sequences expressed in all three Navigators. Fluorescence recovery after photobleaching (FRAP) experiments in COS-7 cells showed that non-plus end associated GFP-Nav1 exhibited slow recovery kinetics, indicating that Nav1 is likely to be part of a high molecular weight complex or cellular structure that diffuses slowly through the cytoplasm (van Haren et al., 2009). Additionally, Nav1 and GFP-Nav2 reportedly associate with F-actin rich areas in the neuronal growth cones and COS-7 cells, respectively (van Haren et al., 2009; Sanchez-Huertas et al., 2020; Powers et al., 2022).

All three Navigators are present in neurite extensions in neurons and neuroblastoma cells, and Nav1 is especially enriched in distal ends of neurites, in growth cones, and at branch points (Martinez-Lopez et al., 2005; Muley et al., 2008; van Haren et al., 2009, 2014; Sanchez-Huertas et al., 2020; Powers et al., 2022). *In vivo*, there is clear enrichment of Nav1 in axonal processes during mouse embryonic brain development (Powers et al., 2022). Similarly in *D. melanogaster*, Sickie is highly expressed in the axons of the mushroom body (Abe et al., 2014). Furthermore, Sickie was also localized to regions of high F-actin in the mushroom body (Abe et al., 2014). Interestingly, all three mammalian Navigators also reportedly showed localization to the centrosome (van Haren et al., 2009).

The localization of the Navigators and the invertebrate homologs in cell types that undergo morphogenesis during development, as well as their subcellular localization to cytoskeletal structures, implicates these proteins as regulators of cytoskeleton-associated functions during development. The next section will review the cellular processes and molecular pathways in which unc-53, Sickie, and the Navigators participate.

## Functions of the Navigators

### Insights from mouse models

There are few published studies using vertebrate models of the Navigators in the whole organism. Nevertheless, some studies have provided insight into the important role of these proteins *in vivo*. For example, *Nav2* hypomorphic mice have been used to uncover its role in the brain (Peeters et al., 2004). Mice hypomorphic for *Nav2* (hypomorphic because the dominant large transcript containing the CH domain was abolished, but the shorter transcript was not), showed reduced body weight and smaller organs, as well as various sensory phenotypes compared to control mice. These sensory defects included deficits in olfaction and pain sensitivity revealed by behavioral tests, as well as a smaller optic nerve revealed histologically. Another pair of studies in *Nav2* hypomorphic mice found defects in cranial nerve development and blood pressure regulation, as well as cerebellar abnormalities and ataxic behavior (McNeill et al., 2010, 2011). Specifically, most *Nav2* hypomorphic cerebella were smaller, and had fewer lobes and foliation compared to wildtype. Furthermore, granule cell migration was impaired in hypomorphic cerebella *in vivo*, and migration and neurite outgrowth defects were observed in cerebellar explants from the knockout mice (McNeill et al., 2011). A more recent paper also observed a reduction of cerebellar size in these mice, including abnormalities in the development of the *VIa* and VIb/VII lobes, as well as a thin corpus collosum, and size reduction in the thalamus and hypothalamus (Accogli et al., 2022). All of these phenotypes demonstrate that Nav2 plays an essential role in neuron migration and morphogenesis of several areas of developing brain.

### Insights from non-rodent models

#### Unc-53 in *Caenorhabditis elegans*

*Unc-53* loss of function mutants in *C. elegans* exhibit defects in multiple organ systems that are associated with cell migration. *Unc-53* mutants were first described in 1974 to have an uncoordinated body motility phenotype (Brenner, 1974), as defined by the “uncoordinated” designation for all *unc* genes. Since that point, *unc-53* and its homologs have been implicated in cellular migration in multiple cell types.

#### Sickie in *Drosophila melanogaster*

In *D. melanogaster*, *sickie* loss of function mutants have been characterized in a few papers, providing insight into the processes in which the Navigators may be involved (Abe et al., 2014; Walkinshaw et al., 2015; Accogli et al., 2022). The first paper describes a mutant that lacks a 510 amino acid region containing a proline rich-region and a coiled-coil domain in one allele, and a transposon inserted into that same region in the second allele (Abe et al., 2014). This mutant displayed abnormalities in brain formation in the central lobe and mushroom bodies, which were attributed to defects in axon growth (Abe et al., 2014). A Drosophila loss of function mutant described in Accogli *et al* (Accogli et al., 2022) introduced a cassette between *sickie* exons 10 and 11 into one allele. The resulting mutant heterozygotes were semi-lethal, and displayed motor defects and heat-induced seizures (Accogli et al., 2022), suggestive of neurological dysfunction. Sickie was also identified as important for labile memory suppression as a component of the active zone in specific dopaminergic neurons (Zhang et al., 2022), implicating the Nav family in memory processes. RNAi knockdown of *sickie* reduced Ca^2+^ influx and dopamine release and identified Bruchpilot, a critical structural protein for the active zone, as a Sickie binding partner (Zhang et al., 2022). This study demonstrates that the Nav family is important for nervous system processes in adult neurons in addition to during development. This concept warrants further investigation in vertebrates.

#### Nav3 in zebrafish

Nav3 loss of function mutants have been investigated in zebrafish (Klein et al., 2011; Lv et al., 2022). Knockdown of *nav3* in zebrafish embryos impeded liver bud formation (Klein et al., 2011), and *nav3* null mutants created *via* CRISPR/Cas9 exhibited morphological and structural defects in the heart, resulting in altered heartbeat intensity in the mutants (Lv et al., 2022). These studies and others implicate the Navigators in the development of multiple organ systems.

## Navigators in neurodevelopmental and neurological disorders: insights from patient case studies

### Navigator 1

*NAV1* is located on human chromosome 1q32.1, a region of the chromosome for which duplications or deletions are associated with instances of neurodevelopmental disorders in patients (Olson et al., 2012; Carter et al., 2016). Symptoms in such patients include developmental delay and impairment in cognitive and motor function. *NAV1* is one of several central nervous system-expressed genes at this chromosomal location; KIF21b and KDM5B are also contained within these regions. KIF21b is a kinesin motor protein, and KDM5B is histone lysine demethylase. Kif21b recently was implicated in a neurodevelopmental disorder characterized by microcephaly and brain malformations (Asselin et al., 2020). While, to date, KDM5B has not been implicated in any disorders, other histone lysine demethylases have been implicated in neurodevelopmental and neurodegenerative disorders (Shi, 2007). Therefore, it is unlikely the loss or duplication of *NAV1* is the sole reason for the symptoms associated with copy number variants (CNVs) at 1q32.1. However, the brain malformations and apparent migration defects observed in these case studies suggest that *NAV1* may contribute to disease phenotypes. Additionally, a small but significant reduction of Nav1 mRNA was observed in the prefrontal cortex of individuals with schizophrenia (Fung et al., 2011). While follow-up is necessary to determine the contribution of Nav1 to schizophrenia pathology, this study suggests Nav1 is involved in multiple neurodevelopmental and neuropsychiatric diseases.

### Navigator 2

Recently, biallelic single point mutations *in NAV2* were identified as disease-causing for a patient with neurodevelopmental delay and diagnoses of cerebellar dysplasia and hypoplasia (Accogli et al., 2022). Researchers detected almost no full-length *NAV2* mRNA or NAV2 protein in patient fibroblasts compared to age-matched controls, although they did identify in immunoblots bands of lower molecular weight, possibly from truncated or degraded fragments of the protein. The patient displayed microcephaly and motor and language delays, and patient fibroblasts had deficient migration *in vitro* (Accogli et al., 2022). This case study marks the first described human disorder caused by mutation in and presumptive loss of a Navigator protein. It thereby highlights the importance of understanding the cellular mechanisms underlying the role of the Navigators in CNS development. Interestingly, multiple single-nucleotide polymorphisms in the *NAV2* gene have been associated with Alzheimer’s Disease risk and age of onset (Wang et al., 2017). While more research needs to be done to confirm the involvement of Nav2 in Alzheimer’s Disease, these studies highlight the potential of Nav2 to contribute to neurodevelopmental and neurodegenerative disease.

### Navigator 3

A recent study of a large cohort of patients with Autism Spectrum Disorder (ASD) identified *NAV3* as a moderate risk gene for ASD (Zhou et al., 2022). Nav3 was described as a rare, inherited loss of function gene with exome-wide significance (Zhou et al., 2022). This finding, along with the other studies cited in this section, highlights the need for further study on all of the Navigator genes and their involvement in neurodevelopmental, neuropsychiatric, and neurodegenerative disorders.

## Cellular roles and mechanisms of the Navigators

Both the invertebrate and vertebrate Navigators are associated with regulating the cytoskeleton during cell migration and morphogenesis. This next section will review the current literature to highlight how the Navigators regulate the cytoskeleton, binding partners that have been implicated, and how Navigators may influence cellular behavior, especially migration and morphogenesis. Table 1 summarizes these reports.

**Table 1:** Proposed cellular function of the navigators based on studies in different organisms.

Organism	Cellular function	Family member	Associated proteins	Reference
*Caenorhabditis elegans*	Distal tip cell migration	Unc-53	abl-1^+^; unc-5^+^; ced-10^#^	Pandey et al. (2018)
*C. elegans*	Excretory cell migration	Unc-53	abi-1^*^	Schmidt et al. (2009)
*C. elegans*	Sex myoblast migration	Unc-53	EGL-15/FGFR^#^; sem-5/GRB2^*^	Stringham et al. (2002)
*C. elegans*	Sensory neuron and motoneuron axon outgrowth	Unc-53	sem-5/GRB2^*^	Stringham et al. (2002)
*Drosophila melanogaster*	Mushroom body axon outgrowth	Sickie	rac-cofilin^#^	Abe et al. (2014)
*D. melanogaster*	Maintain active zone structure	Sickie	Bruchpilot^+*^	Zhang et al. (2022)
Zebrafish	Hepatocyte migration	Nav3		Klein et al. (2011)
Zebrafish	Heart development	Nav3		Lv et al. (2022)
N1E-115 mouse neuroblastoma cells	Neuritogenesis	Nav1	Trio^*^; Rac1^#^	van Haren et al. (2009)
SH-SY5Y human neuroblastoma Cells	Neuritogenesis	Nav2	14–3-3ε^*^	Marzinke et al. (2013)
Rodent Cortex	Neuron migration and orientation	Nav1		Sanchez-Huertas et al. (2020), Powers et al. (2022)
Rodent neurons, SH-SY5Y	Macropinocytosis	Nav1		Powers et al. (2022)
Rodent brain	Cerebellar development cell migration axon outgrowth	Nav2		McNeill et al. (2011), Accogli et al. (2022)

+: genetic interaction *: evidence of direct binding #: implicated cellular pathway.

### Invertebrate Navigators

#### Unc-53 cellular phenotypes

Unc-53 mutants display defects in sex myoblast pathfinding. During migration, myoblasts are guided to the gonads by guidance cues, especially EGL-17/FGF, and the cue is integrated through a pathway involving Sem-5, whose mammalian homolog is the adaptor protein Growth factor bound receptor 2 (GRB2; Stringham et al., 2002), which functions in the Ras signaling pathway. *Unc-53* mutants are defective in the anterior–posterior migration of sex myoblasts, and *sem-*5 mutants show similar sex myoblast migration defects (Stringham et al., 2002). In fact, UNC-53 was shown to bind SEM-5 directly *in vitro* (Stringham et al., 2002).

*Unc-53* is also necessary for distal tip cell (DTC) migration in *C*. *elegans*. DTCs are gonad cells that migrate during the larval stages to form the U-shape gonads. In *unc-53* mutants, the DTCs exhibit polarity reversal and pathfinding defects resulting in abnormal gonads (Pandey et al., 2018). These phenotypes are also seen in *ced-10* and *mig-2* mutants (homologs of Rac and RhoG, respectively), and this paradigm was used to identify other genetic interactors of *unc-53* (Pandey et al., 2018). Double mutants of *unc-53* and either *ced-10* or *mig-2* partially rescued the DTC defects, especially the polarity reversal phenotype, indicating a negative relationship between *unc-53* and *ced-10* or *mig-2*. The Rho/Rac pathway is also involved in Nav1-regulated neurite outgrowth, which will be discussed later (van Haren et al., 2014). *Unc-53* and *abi-1*, a molecule also important in DTC migration, similarly have a negative relationship, as *unc-53:abi-1* double mutants have less severe DTC and gonad phenotypes than either single mutant (Pandey et al., 2018). Though further experimentation is needed to validate the relationships between these proteins in the signaling pathway, these genetic studies highlight the importance of Navigators in cell migration and tissue morphogenesis and demonstrate the interaction of Navigators with other cell migration and/or cytoskeleton-associated molecules to influence cell behavior.

#### Unc-53 in neurite outgrowth

The Navigators and their invertebrate homologs are implicated in neurite outgrowth in their respective organisms. Lack of *unc-53* in *C. elegans* results in impaired axon outgrowth in ALN and PLN neurons (Stringham et al., 2002). The axons in *unc-53* mutants have decreased outgrowth and are misguided. These axons usually travel from the tail to the head of the animal along the anterior–posterior axis, but mutant axons frequently stop before the midbody, and send axonal branches in the dorsal or ventral direction, rather than the anterior (Stringham et al., 2002).

Only a few binding partners and underlying mechanisms of UNC-53 have been identified to date in *C. elegans.* In addition to SEM5, it was shown that Abelson Interactor-1 (ABI-1) directly interacts with UNC-53 at the UNC-53 N-terminus (Pandey et al., 2018). ABI-1 is a protein conserved through vertebrates that is a part of the Wiskott-Aldrich-syndrome-protein (WASP) verproline homologous protein (WAVE) complex. The WAVE complex induces actin nucleation *via* the Arp2/3 complex and is important in neurite outgrowth and growth cone behavior (Stradal et al., 2001; Woodring et al., 2002). This reinforces the notion that cytoskeleton regulation is a key mode of action for UNC-53. ABI-1 also regulates responses to the Abelson tyrosine kinase (Abl), and forms complexes with EPS8 and SOS1, all of which are implicated in various types of trophic factor signaling (Innocenti et al., 2003; Kotula, 2012). UNC-53 and ABI-1 have mostly overlapping expression patterns in the organism, and mutants of each gene display similar phenotypes. These include deficient migration and outgrowth of excretory cells, and defects in axon growth of mechanosensory neurons (Stringham et al., 2002). Additionally, both UNC-53 and ABI-1 are expressed in motoneurons, and mutants of either gene result in dysregulated branching and dorsal outgrowth, and impaired development of a proper neuronal network. Thus, UNC53 and the WAVE complex member ABI-1 converge to regulate motoneuron development in the nematode (Stringham et al., 2002).

Interestingly, 14–3-3ε was identified as a binding partner of mammalian Nav2, and a *C. elegans* mutant of its homolog, *ftt-2*, also displays defects in PLM neuron outgrowth like the *unc-53* mutant (Marzinke et al., 2013). These phenotypes described in *C. elegans* highlight that UNC-53 is important for integrating guidance cues, as cell migration and neurite outgrowth are directed by such cues. Investigations into Sickie and the vertebrate Navigators provide further insight into these molecular processes, as well as the role of the Navigators as integrators of extracellular cues.

#### Sickie in neurite outgrowth

Abe *et al* (Abe et al., 2014) examined the role of Sickie in the formation of the mushroom body, a neuronal structure in the central brain of *D. melanogaster* (Abe et al., 2014). As with *unc-53*, *sickie* is most similar to Nav2. Flies without Sickie had smaller mushroom body axonal lobes, shorter axon branches, and defective ellipsoid bodies (Abe et al., 2014). These phenotypes and further experiments revealed that Sickie acts upstream of cofilin and contributes to axon growth by regulating cofilin through the protein phosphatase slingshot (Abe et al., 2014), although whether this regulation is direct or indirect remains unclear. Slingshot dephosphorylates cofilin in opposition to the Pak-LIM kinase pathway, and subsequently balances actin dynamics, aids in actin recycling, and regulates axon pathfinding. The loss of Sickie in this context results in less active cofilin and, therefore, less cofilin-mediated actin reorganization, which is necessary for proper axon growth (Abe et al., 2014). This further demonstrates the critical role that Navigators can play in regulating actin cytoskeletal dynamics during development.

### Vertebrate Navigators

#### Nav3 in zebrafish

The role of Nav3 in migrating hepatocytes was explored in zebrafish. Nav3 deficient hepatocytes failed to migrate from the endoderm, resulting in smaller livers, and overexpression of Nav3 resulted in increased liver budding (Klein et al., 2011). These studies suggest a gene dosage effect of Nav3 on tissue morphogenesis. Furthermore, Nav3 associated with lamellipodia and filopodia in migrating zebrafish liver cells *in vitro* and *in vivo* (Klein et al., 2011). Fluorescence recovery after photobleaching (FRAP) of Lifeact-RFP *in vitro* also demonstrated that actin polymerization was reduced when Nav3 was knocked down using shRNA (Klein et al., 2011). Interestingly, Nav3 was present in actin protrusions of migrating cells, but this presence was lost upon expression of dominant negative CDC42, and enhanced upon expression of a constitutively active CDC42 (Klein et al., 2011). CDC42 is a Rho GTPase that helps regulate actin formation *via* the Arp2/3 complex (Rohatgi et al., 1999) While there is no evidence thus far of a direct interaction of CDC42 with Nav3 (or any of the Navigators), this experiment reveals another possible mode of action on the cytoskeleton by the Navigators in migrating cells.

#### Cellular mechanisms of Navigator regulation of neuritogenesis

Investigation into the mammalian Navigators, similar to the invertebrate navigators, point to neuritogenesis as a key function, and provides insights into underlying cellular mechanisms. Exogenous expression of all three mammalian Navigators can induce neurite-like extensions in normally non-polarized cells, such as COS-7 cells, demonstrating their capacity to re-organize cytoskeleton and cell periphery and create extensions (van Haren et al., 2009). Nav2 is necessary for neuritogenesis in neuron-like SH-SY5Y cells (Muley et al., 2008; Marzinke et al., 2013), and Nav1 is necessary for neuritogenesis in multiple systems, including neuroblastoma cells and primary hippocampal neurons (van Haren et al., 2014; Powers et al., 2022). However, only a few studies have investigated the underlying cellular and molecular mechanisms of the involvement of the Navigators in these processes. The rest of this section will outline our knowledge to date.

Extracellular cues are integral to proper development in multiple systems, including the nervous system. Nav1 and Nav2 both regulate neurite outgrowth in response to extracellular cues (Martinez-Lopez et al., 2005; Muley et al., 2008; Sanchez-Huertas et al., 2020; Powers et al., 2022), and Nav3 may be activated in response to Wnt2bb in zebrafish (Klein et al., 2011), as Wnt2bb is an early guidance cue for differentiation of liver cells in zebrafish (Ober et al., 2006). While there is currently no evidence of Nav3 responding to guidance cues in neurons, those data demonstrate the capacity of Nav3 to regulate responses to extracellular cues in general. Nav1 and Nav2, have indeed been shown to integrate extracellular cues in neurons. In mouse neuron explant culture, Nav1 is necessary for proper directional neurite outgrowth in response to the attractant cue netrin (Martinez-Lopez et al., 2005; Sanchez-Huertas et al., 2020). However, only a few studies have investigated the underlying cellular and molecular mechanisms of the involvement of the Navigators in neuritogenesis and response to extracellular cues. The rest of this section will outline our knowledge to date.

Nav2 mRNA expression is induced in SH-SY5Y human neuroblastoma cells after all-*trans* retinoic acid (atRA) treatment. In addition, Nav2 expression becomes lower or higher in the developing rat nervous system with atRA deficiency or excess, respectively (Muley et al., 2008), demonstrating that Nav2 expression is sensitive to atRA in multiple systems. Furthermore, neurite outgrowth is impaired in atRA treated Nav2 deficient SH-SY5Y cells (Muley et al., 2008), and neurite outgrowth is impaired also in atRA-plus trophic factor treated Nav1 deficient SH-SY5Y cells (Powers et al., 2022).

In mouse neuron explant culture, Nav1 is necessary for proper directional neurite outgrowth in response to the attractant cue netrin (Martinez-Lopez et al., 2005, Sanchez-Huertas et al., 2020). Additionally, Nav1 is important for cortical neuron migration (Sanchez-Huertas et al., 2020) and leading process orientation *in vivo* (Powers et al., 2022) an orientation event that is necessary for directional migration that establishes the six layered neocortex in mammals (Marín et al., 2010). These studies demonstrate that the Navigators are important across species in regulating the cellular and molecular responses to extracellular cues to direct cell migration and neuritogenesis in multiple tissues and cellular contexts, suggesting this may be a shared and critical role among the Navigators.

#### Subcellular mechanisms of neuritogenesis regulation by Navigators

A yeast two-hybrid screen and subsequent biochemical experiments identified 14–3-3ε and 14–3-3β as direct binding partners *via* amino acids 761–960 of Nav2 (Marzinke et al., 2013). 14–3-3ε as a Nav2 binding partner is especially interesting because of its established importance in early neural development. In fact, Miller-Dieker Syndrome, a neurodevelopmental disorder characterized by lissencephaly, can be caused by a loss of 14–3-3ε (Toyo-oka et al., 2003). The multiple 14–3-3 family proteins are essential regulatory and adaptor proteins in a variety of signaling pathways, and 14–3-3ε has been shown to regulate the degradation of δ-catenin to promote neuronal migration (Toyo-oka et al., 2003, 2014). Co-expression of exogenous Nav2 and 14–3-3ε yielded overlapping localization near microtubules in the cell body and the neurite. Reduction of 14–3-3ε in SH-SY5Y cells resulted in impaired atRA-induced neurite outgrowth, similar to silencing of Nav2 (Marzinke et al., 2013).

The identification of the Navigators as microtubule binding proteins (Martinez-Lopez et al., 2005; van Haren et al., 2009; Marzinke et al., 2013), and + TIP proteins specifically (Martinez-Lopez et al., 2005; van Haren et al., 2009), led to interest in their influence on microtubules and how that may affect their role in neuritogenesis. Overexpression of all three Navigators promoted microtubule bundling (van Haren et al., 2009). A recent study revealed that microtubules in LLCPK-α cells overexpressing Nav1 had shorter depolymerization events compared to control cells, and microtubules also spent more time pausing (Sanchez-Huertas et al., 2020). Furthermore, Nav1 promoted microtubule persistence in the growth cone periphery (Sanchez-Huertas et al., 2020), consistent with observations from our own lab demonstrating that Nav1 is more highly concentrated on plus ends that are in the periphery of the growth cone compared to the central domain of the growth cone (Powers et al., 2022). These data together suggest that Nav1 prevents microtubule catastrophes, preferentially associates with plus ends in the growth cone periphery, and affects microtubule dynamics in the neuronal growth cone.

The binding partners and molecular mechanisms underlying the regulation of the cytoskeleton by Nav1 are of particular interest as Nav1 lacks the actin-associated calponin homology domain present in Nav2 and Nav3. One binding partner identified in N1E mouse neuroblastoma cells is Trio (van Haren et al., 2014), a Rho and CDC42 guanine exchange factor (GEF) that regulates actin dynamics *via* the Rho/Rac pathway and is important in neurite outgrowth (Estrach et al., 2002; Briançon-Marjollet et al., 2008). Nav1 associates with Trio at the microtubule plus end *via* the Nav1 microtubule binding domain, and this interaction is necessary for neurite outgrowth *via* activation of Rac1 (van Haren et al., 2014). Interestingly, the authors showed that GFPNav2 also binds Trio and EB1, and that GFPNav2-induced neurite outgrowth was abolished after addition of a dominant-negative Trio that binds EB1 but has no GEF activity (van Haren et al., 2014). These data suggest that Nav1 and Nav2 share similar functions and may operate in the same pathway(s) to promote neuritogenesis.

A recent study offered evidence that Nav1 may directly bind F-actin despite its lack of a CH domain. Sanchez-Huertas *et al* found that Nav1 can mediate cross-linking to microtubules and F-actin *in vitro* (Sanchez-Huertas et al., 2020). Depletion of Nav1 caused increased growth cone area and a higher density of filopodia with more extension events, though there was no change in filopodial length. These data suggest Nav1 influences actin dynamics in the growth cone.

#### Nav1 in macropinocytosis

**Figure 7:**
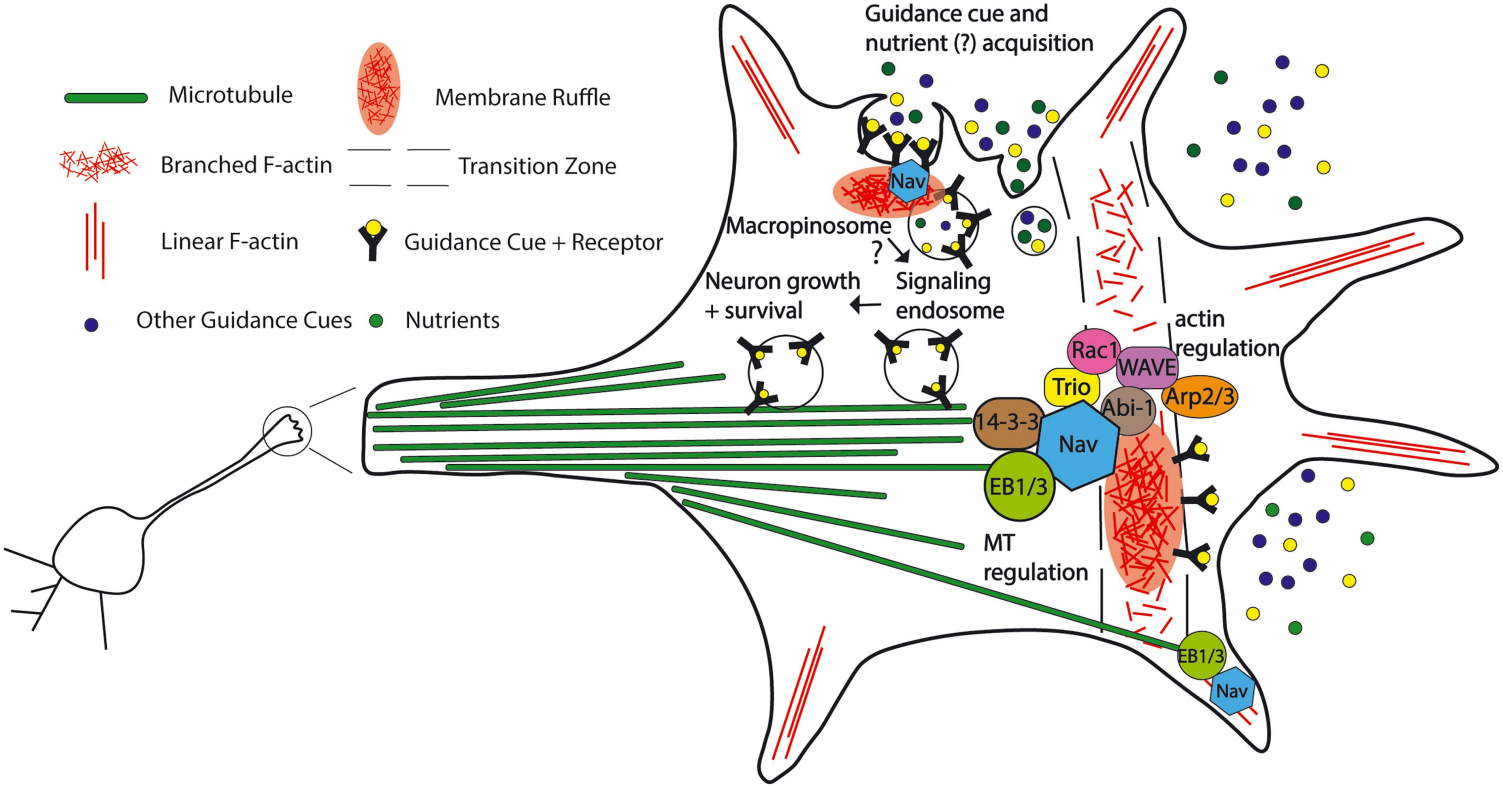
Proposed model for the functions and protein interactions of the Neuron Navigators in the neuronal growth cone. A subset of the Navigator population localizes to the plus-tip of microtubules *via* binding to end binding protein, cross-links microtubules and actin, and influences cytoskeleton dynamics in the growth cone. Nav1 promotes actin-rich membrane ruffles in the transition zone of the growth cone. This may be *via* binding to other cytoskeleton-associated molecules such as Trio, 14–3-3 family proteins, and members of the WAVE complex. Nav2 and Nav3 may also influence membrane ruffles *via* their putative actin-binding domain bind to cytoskeleton-associated molecules, and influence neuritogenesis. Nav1 also promotes macropinocytosis, a non-selective fluid-phase endocytosis in the growth cone, including internalization of guidance cues and their receptors, possibly leading to downstream neuron growth regulation *via* signaling endosomes. Growth cone macropinocytosis may also contribute to extracellular nutrient and metabolite acquisition, as seen in cancer cells. (See text for details and references).

Adding further evidence to the regulation of F-actin by Nav1, Powers *et al* (Powers et al., 2022) demonstrated that Nav1 promotes F-actin rich membrane ruffles in the growth cone, which gather within the transition zone. Membrane ruffles are patches of concentrated F-actin whose role in the growth cone is not well delineated but are involved in cell migration, endocytosis, and morphogenesis in other cell types (Ridley et al., 1992; Radhakrishna et al., 1999; Borm et al., 2005). Membrane ruffles also participate in membrane recycling in growth cones (Bonanomi et al., 2008). Nav1 promotes macropinocytosis at these F-actin rich membrane ruffles, and furthermore, Nav1 promoted membrane accumulation at the ruffles, demonstrating a novel connection of Nav1 and the plasma membrane (Powers et al., 2022). Interestingly, loss of Nav1 in SH-SY5Y cells induces membrane blebbing, implying a disconnection between the plasma membrane and cortical F-actin. This further supports a role for Nav1 in regulating the dynamics of the cytoskeleton and the plasma membrane (Powers et al., 2022). Notably, Nav1 also promoted in the growth cone the internalization of TrkB, the receptor for brain-derived neurotrophic factor (BDNF; Powers et al., 2022), a neurotrophin that regulates neuritogenesis and synaptic function (Sonoyama et al., 2020; Zagrebelsky et al., 2020). Endocytosis of the BNDF-TrkB complex leads to downstream signaling events that promote cell survival and neurite growth (Cheung et al., 2007; Zhou et al., 2007; Zheng et al., 2008; Xu et al., 2016), and so the observation that Nav1 promotes internalization of this receptor provides new insight into why Nav1 is necessary for proper neuritogenesis (Figure 7). This observation also demonstrates that Nav1 is involved with extracellular cues beyond netrin during neuron development. Furthermore, it has been suggested that Trk-containing macropinosomes are marked by the protein Pincher to avoid degradation, and thereby perpetuate long-range signals in response to neurotrophins to promote neural growth (Valdez et al., 2005, 2007). Transduction of a variety of extracellular guidance cues may be a general characteristic of the Navigator molecules, possibly acting *via* fluid-phase uptake mechanisms like macropinocytosis (Figure 7).

## Navigators in cancer

Several studies have pointed to a role for the Navigators in various cancers, most of them highlighting differential expression of one of the Navigators in association with cancer cells or cancer progression, including breast cancer, colorectal cancer and uterine sarcoma (Li et al., 2010; Carlsson et al., 2012, 2013; Cohen-Dvashi et al., 2015; Tan et al., 2015; Davidson et al., 2017; Hu et al., 2019; Uboveja et al., 2020). In particular, Nav2 was shown to promote invasion of colorectal cancer cells (Ishiguro et al., 2002; Tan et al., 2015). These studies report that Nav2 is highly expressed in colorectal cancer cells, and that Nav2 promotes migration and invasion of cancer cells, and therefore overexpression of Nav2 likely promotes metastasis (Ishiguro et al., 2002, Tan et al., 2015). These observations are interesting in light of the discovery of Nav1 involvement in macropinocytosis (Powers et al., 2022), as cancer cells use macropinocytosis to obtain nutrients to drive rapid cell proliferation (Recouvreux and Commisso, 2017; Zhang and Commisso, 2019; Xiao et al., 2021). While the Navigators are apparently not uniformly either upregulated or downregulated in cancer, the differential expression of Navigators across different types of cancers and a potential connection to macropinocytosis presents an interesting area for further study.

## Conclusion and future directions

Many outstanding questions remain regarding the functions, mechanisms, and similarities and differences among the Navigators, as well as their roles across the multiple tissues in which they are expressed (Maes et al., 2002). Investigating the function of all three Navigators in different tissues and comparing functions across tissues and between Navigators will give insight into their specific roles. Tissue-specific single knockouts of the Navigators and comparison of phenotypes will be necessary to assess the similarities and differences.

The conservation of the AAA+ domain from invertebrates through vertebrates, possibly stemming from early metazoans, suggests this is a defining feature of the Navigator family. However, little is known about the relevance of the AAA+ domain to the Navigators’ cellular roles. *In vitro* experiments investigating the nucleoside triphosphatase activity of this domain are lacking. Identification of substrates for the AAA+ activity, and cellular experiments with mutated AAA+ domains to test the contribution of this domain to Navigator function would begin to answer important questions. Similar structure–function experiments investigating the other functional protein domains in the Navigators will provide additional insights. Curiously, as noted above, amino acid sequence suggests that Navigators belong to the McrB family within the helix-2 insert clade (Iyer et al., 2004), which, unlike the majority of AAA+ proteins, hydrolyze GTP rather than ATP as their nucleoside triphosphatase activity (Nirwan et al., 2019). However, neither ATPase or GTPase activity for the Navigators has yet been experimentally confirmed. Although some eukaryotes contain members of this clade (Iyer et al., 2004), the best characterized member is critical in prokaryotic DNA restriction modification. McrB functions in bacteriophage defense as the GTP hydrolyzing subunit of the *E. coli* McrBC restriction system (Nirwan et al., 2019). McrB forms hexamers in the presence of GTP and binds to the endonuclease subunit McrC to perform DNA translocation and cleavage (Panne et al., 1999; Nirwan et al., 2019). It is unclear whether and how this ancestral function aligns with the previous identification of Nav2/HELAD1 as a DNA helicase (Ishiguro et al., 2002). According to current knowledge, the Navigators are mostly concentrated within cytoplasmic regions of cells. Nevertheless, it is too soon to rule out a function associated with nucleic acids. Our protein domain analysis (Figure 1) suggests a possible nuclear trafficking of the Navigators due to the presence of one or more nuclear localization signals. A previous study also reported Nav3/POMFIL1 immunoreactivity at nuclear pores (Coy et al., 2002). Investigating the potential nuclear functions of the Navigators could lead to discovery of yet undescribed functions of this protein family.

Most AAA+ proteins form a hexameric ring with a central pore that mediates substrate translocation driven by the motor-like NTPase activity (Nirwan et al., 2019; Puchades et al., 2020; Khan et al., 2022). It is an outstanding question whether the Navigators form hexamers, and whether they might interact to form hetero-oligomers comprised of multiple Navigators. Therefore, determining whether Navigators interact with one another, determining their native structure, and identifying their key substrate(s) would yield critical insights into Navigator function within the cell.

Finally, to date there have been no targeted investigations of the potential redundancy or interaction among the Navigator proteins, despite the fact that they are expressed in many of the same tissues (Maes et al., 2002). While knockout or knockdown experiments on a single Navigator have been performed, experiments using double or triple knockouts would aid in understanding any redundancy or cooperation among the Navigators. This line of inquiry would likely lead to yet unexplored areas of Navigator biology.

From nematodes to humans, the Navigators and their homologs have proven to be essential for proper development of multiple tissues, including the mammalian nervous system. The Navigators likely integrate cellular responses to multiple extracellular guidance cues in different cell types, and coordinate the cytoskeletal response to these cues *via* both microtubules and actin filaments, in conjunction with membrane trafficking (Figure 7). Based on the discovery that Nav1 promotes macropinocytosis in neurons (Powers et al., 2022) and the similar domain structure of all the Navigators, we propose that uptake of fluid-phase cues and/or downregulation of cell surface receptors could represent general mechanisms that explain the function of the Navigators in cell migration and guidance, as well as cancer. Cancer cells use macropinocytosis to obtain extra nutrients and metabolites (Commisso et al., 2013; Qian et al., 2014; Palm et al., 2015; Recouvreux and Commisso, 2017), suggesting the possibility of a similar mechanism during the energy-demanding process of neuronal outgrowth. The ancient evolutionary origins of fluid-phase uptake mechanisms like macropinocytosis (King and Kay, 2019) aligns with the idea that macropinocytosis may be crucial to multiple biological processes. It is also likely that the Navigators influence morphogenesis and migration *via* interaction with other signaling molecules that directly or indirectly affect cytoskeletal dynamics (Figure 7). The recent identification of a patient with a neurodevelopmental disorder attributable to the loss of Nav2 (Accogli et al., 2022) underscores the urgency to characterize the molecules and the cellular processes in which the Navigators participate, which will further our understanding of human health and disease.

## Author contributions

RP: conceptualization, writing—original draft version, writing—reviewing and editing, figure preparation. RH: conceptualization, writing—original draft version, writing—reviewing and editing, figure preparation. SH: conceptualization, writing—reviewing and editing, figure preparation, supervision. All authors contributed to the article and approved the submitted version.

## Funding

This work was supported by National Institutes of Health (NIH) grants NS37311 and MH087823 and was a component of the National Cooperative Reprogrammed Cell Research Groups (NCRCRG) to study mental illness which was supported by NIH grant U19 MH107367.

## Conflict of interest

The authors declare that the research was conducted in the absence of any commercial or financial relationships that could be construed as a potential conflict of interest.

## Publisher’s note

All claims expressed in this article are solely those of the authors and do not necessarily represent those of their affiliated organizations, or those of the publisher, the editors and the reviewers. Any product that may be evaluated in this article, or claim that may be made by its manufacturer, is not guaranteed or endorsed by the publisher.
