# Supplementary figures and images for: The Neuron Navigators: Structure, function, and evolutionary history
Source: Front Mol Neurosci. 2023 Jan 12;15:1099554. doi: 10.3389/fnmol.2022.1099554 (PMC9877351; doi:10.3389/fnmol.2022.1099554)

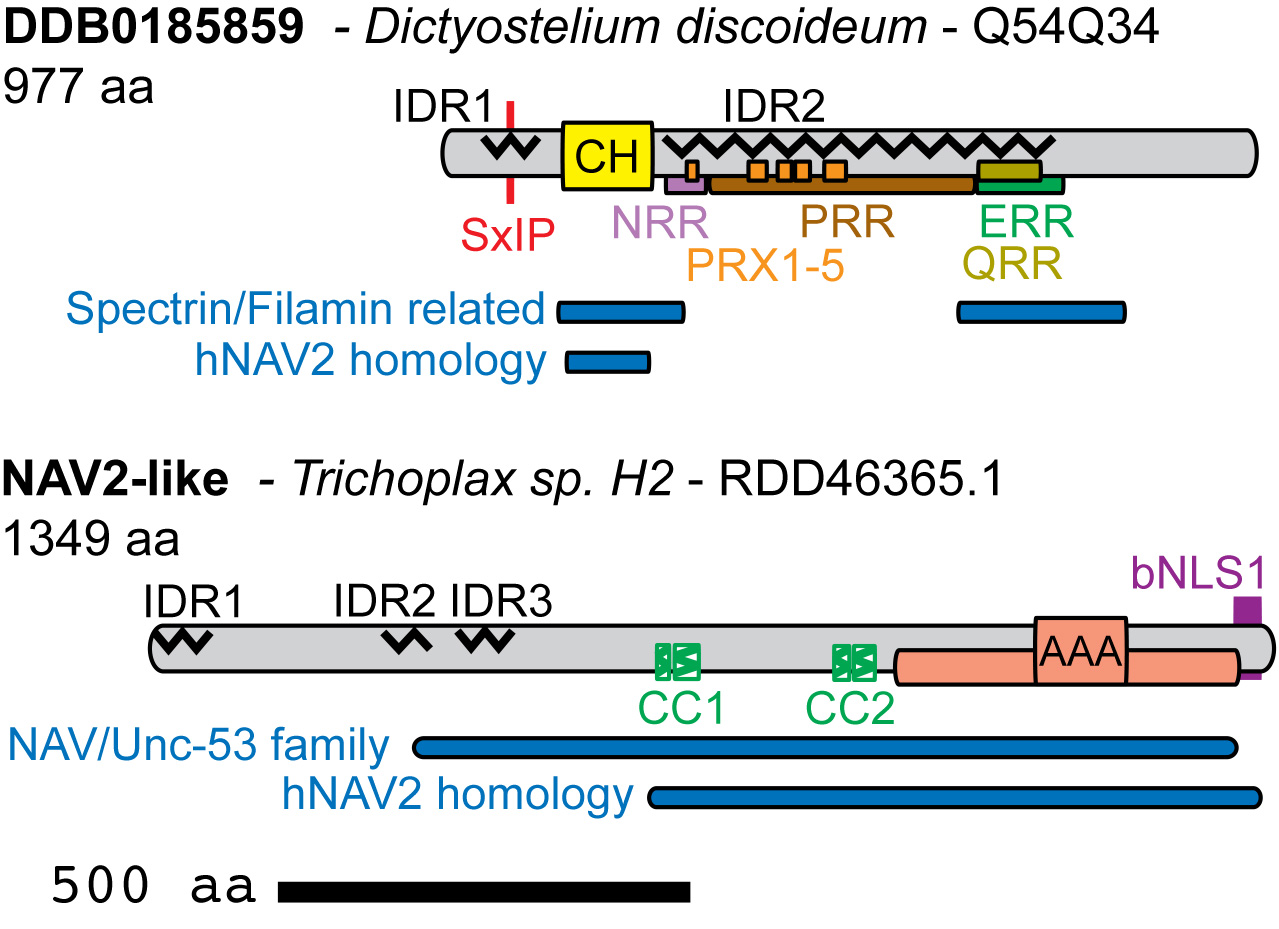

Supplement: Supplementary file 7 [file Image_1.JPEG]
